# Early microbial markers of periodontal and cardiometabolic diseases in ORIGINS

**DOI:** 10.1038/s41522-022-00289-w

**Published:** 2022-04-20

**Authors:** Clarisse Marotz, Rebecca Molinsky, Cameron Martino, Bruno Bohn, Sumith Roy, Michael Rosenbaum, Moïse Desvarieux, Melana Yuzefpolskaya, Bruce J. Paster, David R. Jacobs, Paolo C. Colombo, Panos N. Papapanou, Rob Knight, Ryan T. Demmer

**Affiliations:** 1 Department of Pediatrics, University of California, San Diego, La Jolla, CA USA; 2 Division of Epidemiology and Community Health, School of Public Health, University of Minnesota, Minneapolis, MN USA; 3 Bioinformatics and Systems Biology Program, University of California, San Diego, La Jolla, CA USA; 4 Center for Microbiome Innovation, University of California, San Diego, La Jolla, CA USA; 5 Department of Epidemiology, Mailman School of Public Health, Columbia University, New York, NY USA; 6 Division of Molecular Genetics, Departments of Pediatrics and Medicine, Columbia University, New York, NY USA; 7 Division of Cardiology, Department of Medicine, New York Presbyterian Hospital, Columbia University, New York, NY USA; 8 Department of Molecular Genetics, The Forsyth Institute, Cambridge, MA USA; 9 Department of Oral Medicine, Infection, and Immunity, Harvard School of Dental Medicine, Boston, MA USA; 10 Division of Periodontics, Section of Oral, Diagnostic and Rehabilitation Sciences, Columbia University, New York, NY USA; 11 Department of Computer Science & Engineering, University of California, San Diego, La Jolla, CA USA

**Keywords:** Clinical microbiology, Policy and public health in microbiology, Microbiome, Bacteriology, Applied microbiology

## Abstract

Periodontitis affects up to 50% of individuals worldwide, and 8.5% are diagnosed with diabetes. The high-comorbidity rate of these diseases may suggest, at least in part, a shared etiology and pathophysiology. Changes in oral microbial communities have been documented in the context of severe periodontitis and diabetes, both independently and together. However, much less is known about the early oral microbial markers of these diseases. We used a subset of the ORIGINS project dataset, which collected detailed periodontal and cardiometabolic information from 787 healthy individuals, to identify early microbial markers of periodontitis and its association with markers of cardiometabolic health. Using state-of-the-art compositional data analysis tools, we identified the log-ratio of *Treponema* to *Corynebacterium* bacteria to be a novel Microbial Indicator of Periodontitis (MIP), and found that this MIP correlates with poor periodontal health and cardiometabolic markers early in disease pathogenesis in both subgingival plaque and saliva.

## Introduction

The human oral cavity hosts hundreds of unique microbial taxa. Within the oral cavity, there are multiple distinct niches that contain different compositions of microbial taxa. For example the supra- and subgingival tooth surface, tongue, cheek, and roof of mouth each harbor consistently distinct microbial communities^1^. In the context of severe periodontal disease, the composition of microbial taxa in the supra and subgingival plaque undergo dramatic changes^2–4^. Periodontitis-associated subgingival biofilms often contain a climax community dominated by *Porphyromonas gingivalis*, *Treponema denticola*, and *Tannerella forsythia*, referred to as the ‘red complex’^5^. The red complex has been studied in depth for its ability to negatively affect host physiology through virulence factors and expedite gingival deterioration in severe diseases. However, less is known about the early microbial markers of periodontitis and when compositional changes in plaque biofilms occur relative to disease onset.

Periodontal disease affects nearly half the global population^6^ and is one of the leading causes of tooth loss^7^. This disease has two primary stages, gingivitis and periodontitis. Gingivitis is the early stage of gum disease where the gingival soft tissue becomes swollen and may bleed upon provocation due to the presence of vasculitis and a local inflammatory infiltrate secondary to bacterial challenge^8^. With time, inflammation results in a deepening of the periodontal pocket, further expanding the ecological niche enabling greater plaque accumulation and host response to pathogens in the biofilm. Left unresolved, the early stages of periodontitis occur when the local inflammatory processes induce a progressive loss of tooth-supporting tissues and eventually tooth loss.

There is strong evidence for a link between oral health and cardiometabolic health^9–13^. While it has long been recognized that individuals with diabetes are at higher risk for periodontitis, recent evidence suggests that adverse oral microbial exposures that underlie periodontitis might also contribute to the etiology of cardiometabolic diseases, including diabetes. Few studies have investigated the relationship between oral microbial communities and markers of both periodontal disease and diabetes risk. Identifying microbial signatures that emerge early in disease could help elucidate potential shared microbial etiology of periodontitis and cardiometabolic disease.

Next-generation sequencing has enabled the high-throughput collection of detailed microbial information from thousands of samples. However, this data is inherently compositional, meaning that the results provide information only on microbial relative abundance rather than absolute abundance^14^. Therefore, care must be taken to avoid false positive or negative findings in these datasets, particularly because total microbial load can vary largely among subgingival plaque samples. In this analysis, we use a suite of compositional data analysis tools to identify differentially abundant bacteria in relation to measures of periodontal disease and early biomarkers of cardiometabolic disease.

We applied these tools to a subset of data collected in the Oral Infections, Glucose Intolerance and Insulin Resistance Study (ORIGINS)^15^. This dataset contains information from a large group of individuals free of clinical cardiometabolic disease, including a comprehensive periodontal examination, quantitative cardiometabolic markers, as well as 16 S rRNA gene amplicon sequencing from saliva and subgingival plaque from both healthy and diseased sites, processed separately, which allowed us to look for site-specific microbial markers that could be early indicators of disease.

## Results

### Overview of the cohort

**Fig. 1:**
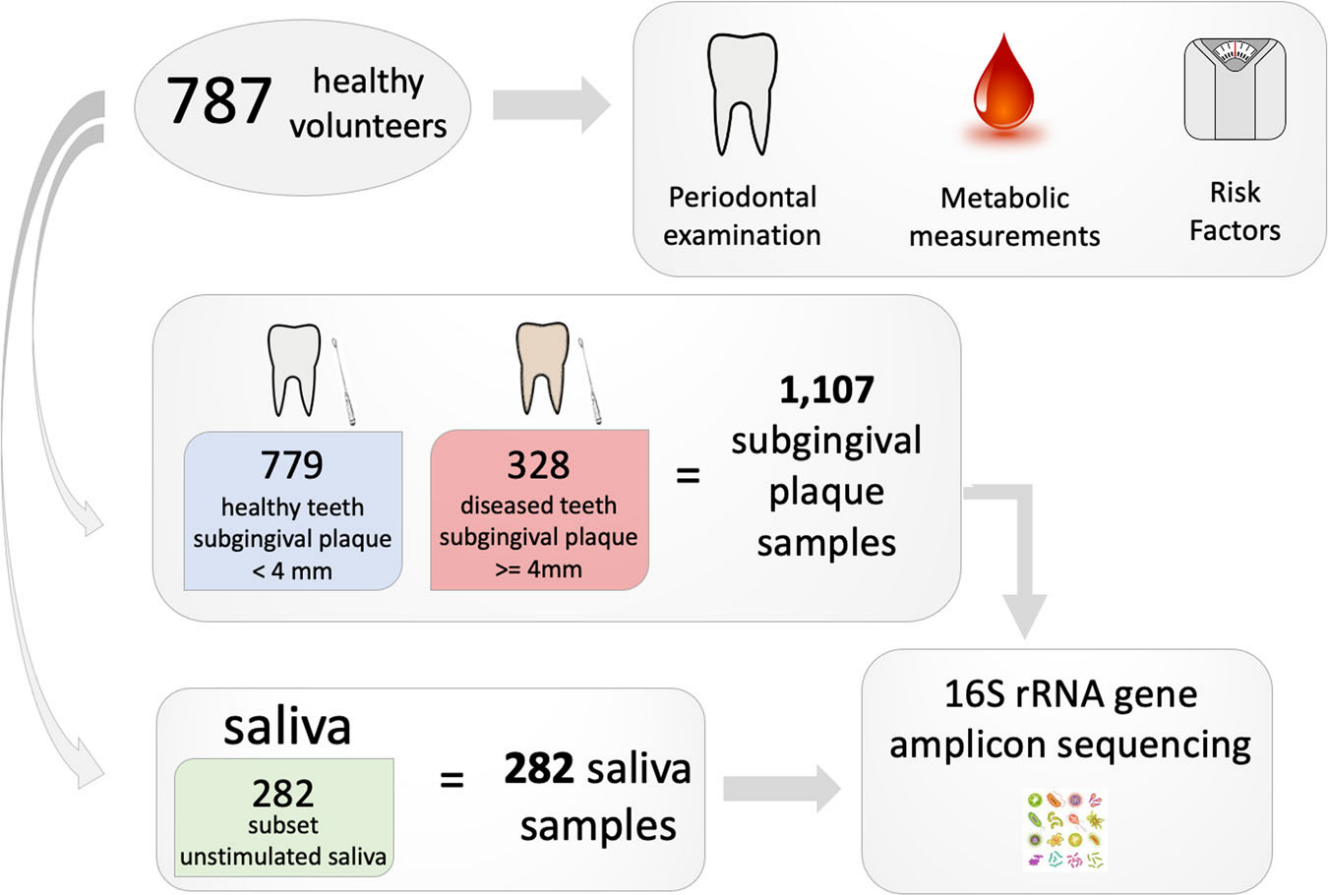
Experimental design. 787 healthy volunteers were recruited to participate in the ORIGINS project. Each participant underwent an extensive periodontal examination, metabolic assessment, and completed standard questionnaires assessing demographic and risk factor information. Subgingival plaque samples were collected from teeth with periodontal pockets <4 mm depth (healthy) and teeth with periodontal pockets ≥ 4 mm depth (diseased) where applicable. In parallel, unstimulated saliva were collected and processed for a subset of individuals. In total, 16 S rRNA gene amplicon sequencing data from 1107 subgingival plaque samples and 282 saliva samples was generated for analysis.

Wave 2 of the ORIGINS project recruited 800 participants, 787 of which had complete data collection for the current analyses. Each participant underwent extensive periodontal examination and metabolic measurements as previously described^15^. The subgingival plaque was collected from healthy (‘shallow’ periodontal pocket <3 mm) and diseased sites (‘deep’ periodontal pocket ≥ 4 mm) as applicable following a standardized protocol totaling 1107 plaque samples (Fig. 1). Saliva samples were collected in parallel and processed for a subset of 282 participants. Both saliva and subgingival plaque samples were processed for 16 S rRNA gene amplicon sequencing. The average full-mouth pocket depth (PD) was 1.90 mm, the average attachment loss was 0.59 mm, and 0.31% of sites exhibited bleeding on probing (BOP). 71%, 26%, and 2% of participants had no or mild periodontitis, moderate periodontitis or severe periodontitis, respectively, according to the CDC/AAP definition. As expected, the prevalence and relative abundance of canonical subgingival plaque pathogens from the red and orange complex^5^ were higher in deep versus shallow periodontal pockets (Table S1).

### Periodontal pocket depth is strongly associated with microbial diversity

**Fig. 2:**
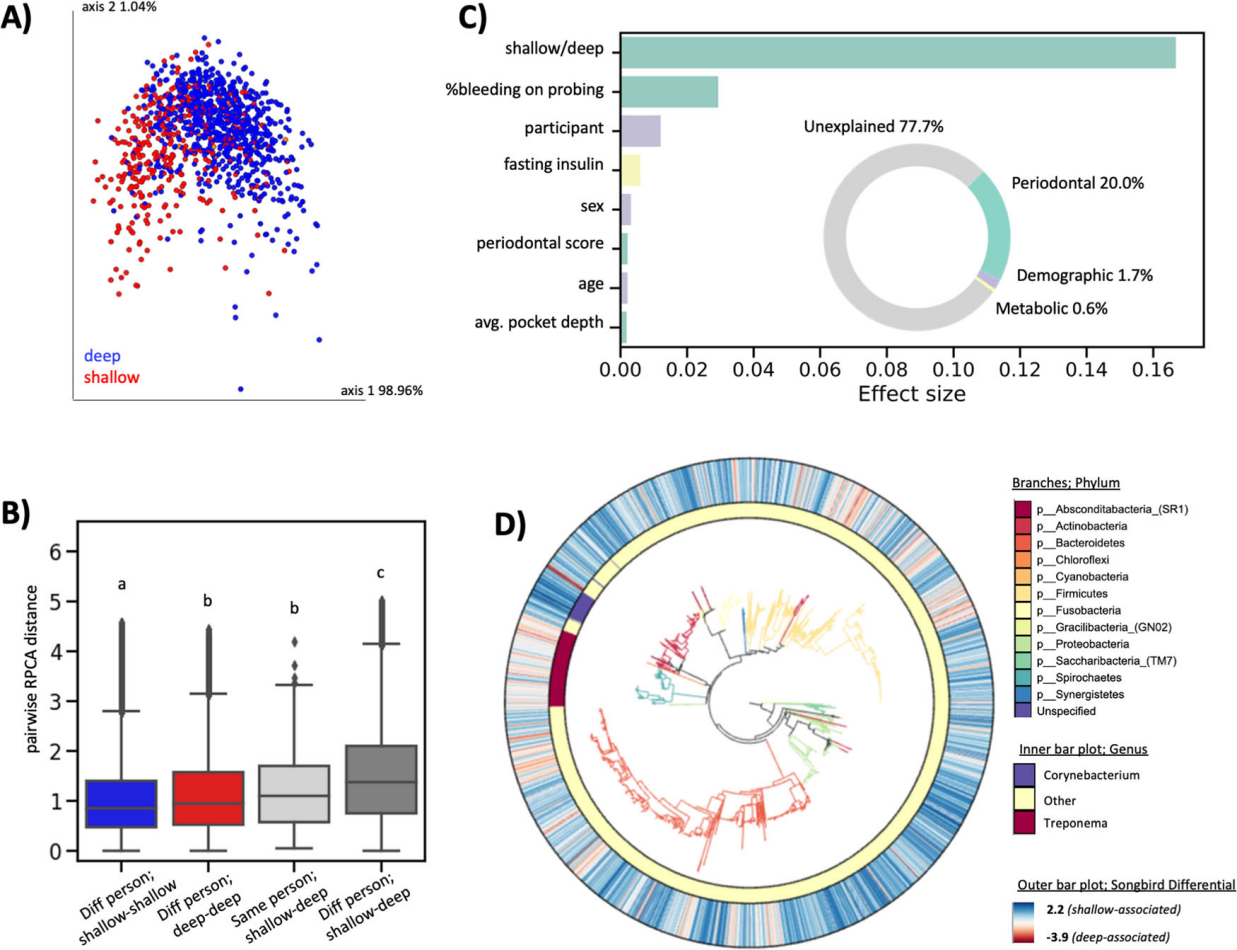
Beta-diversity and redundancy analysis in subgingival plaque. **A** RPCA colored by periodontal pocket depth. Permanova pseudo-F statistic = 397.062, *p*-value < 0.001. **B** RPCA distance among pairwise samples; Subgingival plaque samples from shallow periodontal pockets of different people (*n* = 308,505 pairs), subgingival plaque samples from deep periodontal pockets samples of different people (*n* = 54,285 pairs), subgingival plaque samples from shallow versus deep periodontal pockets from the same person (*n* = 322 pairs), subgingival plaque samples from shallow versus deep periodontal pockets from different people (*n* = 259,058 pairs). Each group is significantly different from all other groups (one-way ANOVA with Tukey’s multiple corrections, *p* < 0.05). The box shows the quartiles of the dataset while the whiskers extend to show the rest of the distribution, except for points that are determined to be “outliers” using a method that is a function of the inter-quartile range. **C** Redundancy analysis (RDA) estimates the percent microbial diversity explained by each variable. Inset donut chart sums effect sizes by category; periodontal variables explained the majority of microbial variation (20.0%), followed by demographic variables (1.7%) and metabolic variables (0.6%). **D** Empress plot displaying ASV-level phylogeny with branches colored by phylum. Outer bar plot represents songbird differentials on a color scale where high values (blue color) are taxa associated with health, and low values (red color) are associated with disease. Inner bar plot highlights features from the genera *Corynebacterium* (blue) and *Treponema* (red), which have high and low Songbird differentials, respectively.

We used the robust Aitchison Principal Components Analysis (RPCA) method^16^, which accounts for the inherent sparsity and compositionality of next-generation sequencing experiments^14^, to assess beta-diversity across subgingival plaque samples. The first axis of separation showed distinct clusters of subgingival plaque collected from shallow versus deep periodontal pockets (Fig. 2A). Interestingly, the microbial composition of subgingival plaque samples from shallow periodontal pockets from different individuals were more similar to each other than the microbial composition of subgingival plaque samples from a shallow and deep periodontal pocket in the same person (Fig. 2B). This finding was only identified using RPCA and not other beta-diversity metrics (including weighted and unweighted UniFrac^17^, Jaccard, and Bray–Curtis), highlighting the ability of this tool to identify novel beta-diversity patterns that accord with expected patterns from prior work reporting consistent patterns of microbial dysbiosis in periodontal subgingival plaque samples^16,18,19^.

We performed an effect size redundancy analysis (RDA) to determine which factors explained the variation in microbial composition across samples^20^. Thirteen non-redundant factors were included in the RDA including, six periodontal metrics (shallow or deep periodontal pocket depth, percent of sites bleeding on probing, full-mouth classification of periodontal status using the CDC/AAP periodontitis definition, average whole-mouth pocket depth, average whole-mouth attachment loss, and percent of sites with attachment loss >3), three demographic factors (participant, sex, and age), three metabolic factors (fasting insulin, prediabetes status, and average systolic blood pressure) and one lifestyle factor (tobacco-smoking status). Of these thirteen factors, eight were found to have a significant effect size (*p*-value < 0.05).

The factor which explained the most variation was the disease status of the site adjacent to plaque sampling (i.e., a deep or shallow periodontal pocket (Fig. 2C)). Overall, periodontal metrics accounted for most of the explained variance (20%) followed by demographic factors (1.7%) and lastly metabolic factors (0.6%). Tobacco smoking did not have a significant effect on subgingival plaque microbial composition in this analysis (87% of the participants reported never smoking).

The multinomial regression tool Songbird^21^ was used to identify differentially abundant microbes in shallow versus deep periodontal pockets. Each amplicon sequence variant (ASV) was assigned a differential where higher scores reflect relative enrichment in shallow periodontal pockets, and low scores reflect relative enrichment in deep periodontal pockets (Table S2). The phylogenetic relationship among these ASVs and their associated Songbird differentials were visualized with EMPress^18^ (Fig. 2D). ASVs from the genus *Treponema* tended to have low (disease-associated) differentials, while ASVs from the genus *Corynebacterium* tended to have high (health-associated) differentials.

### Identifying an early MIP in subgingival plaque

**Fig. 3:**
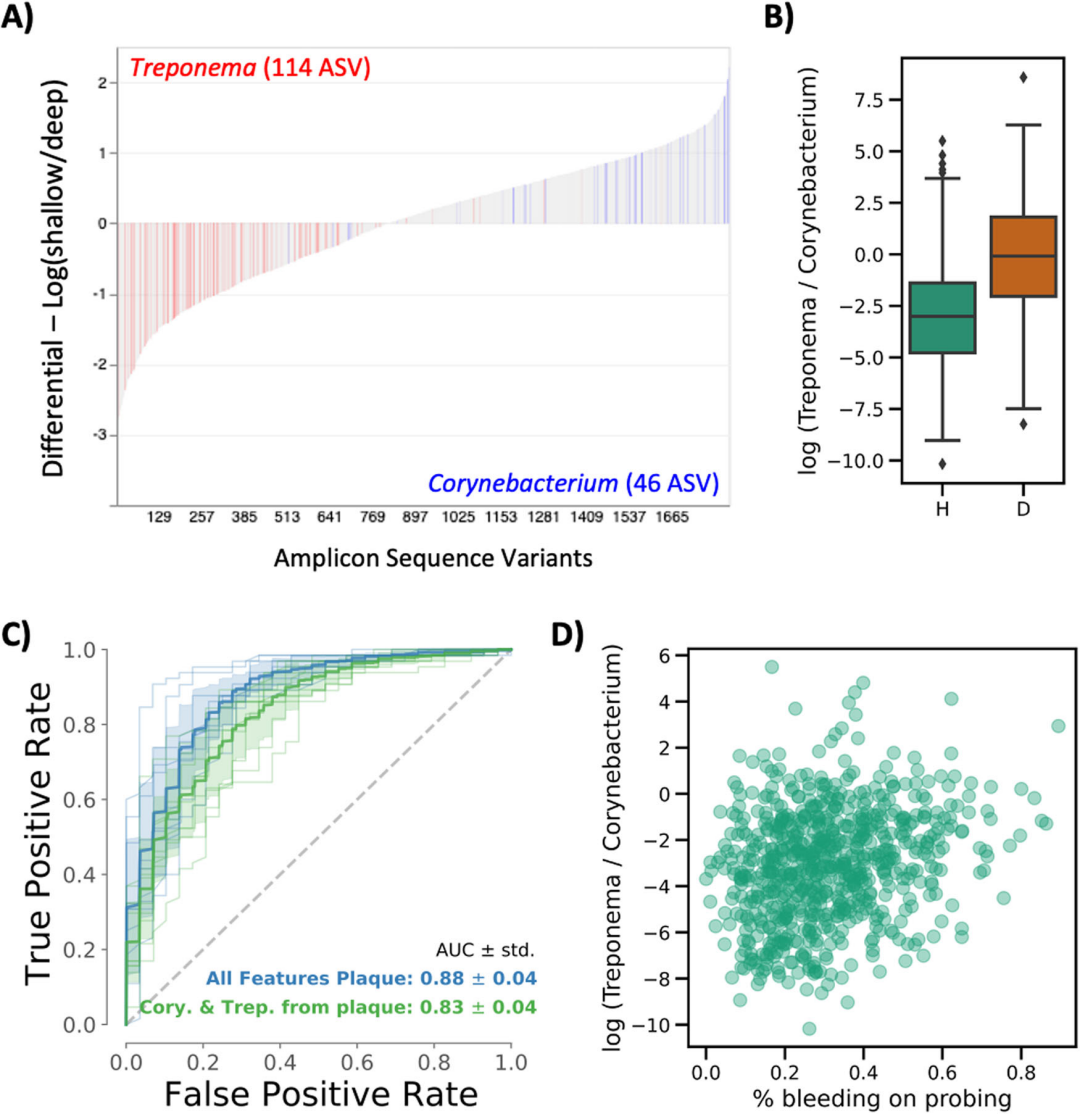
The ratio of Treponema: Corynebacterium is an early microbial indicator of periodontal disease (MIP) in subgingival plaque. **A** Differential ranking with Songbird revealed that *Treponema* sequences in subgingival plaque were associated with deep periodontal pockets, whereas *Corynebacterium* sequences were associated with shallow periodontal pockets. **B** The log-ratio of *Treponema*:*Corynebacterium* significantly distinguishes shallow (H = healthy sites) from deep (D = diseased sites) periodontal pockets and is used as a Microbial Indicator of Periodontal Disease (MIP). The box shows the quartiles of the dataset while the whiskers extend to show the rest of the distribution, except for points that are determined to be “outliers” using a method that is a function of the inter-quartile range. **C** ROC curve displaying the accuracy of a Random Forest classifier trained on the full dataset (blue) versus trained only on *Treponema* and *Corynebacterium* sequences and log-ratio (green) shows similar accuracy at predicting shallow versus deep periodontal pocket depth. **D** In plaque collected from shallow (healthy) subgingival pockets (*n* = 779), MIP was positively correlated with the percent of sites bleeding on probing (Pearson correlation = 0.243, *p-value* = 8.06e^−12^), indicating that microbial changes occur in plaque before clinically meaningful pocketing.

To further characterize these health- and disease- associated ASVs, we plotted the Songbird differentials from each ASV using the interactive tool Qurro^22^. As observed in the phylogenetic analysis, *Treponema* ASV differentials were more associated with deep periodontal pockets, whereas ASVs aligned to the genus *Corynebacterium* were more associated with shallow periodontal pockets (Fig. 3A). To generate a microbial indicator of periodontitis (MIP), we used *Corynebacterium* as a ‘reference frame’ and calculated the log-ratio of all *Treponema* counts to all *Corynebacterium* counts. This MIP was significantly higher in deep compared to shallow periodontal pockets (paired *T*-test < 0.0001) and revealed that the ratio of *Treponema* to *Corynebacterium* is roughly even in deep periodontal pockets, whereas the ratio in shallow periodontal pockets is heavily skewed towards *Corynebacterium* (Fig. 3B). Importantly, because these are relative abundance data, we cannot conclude whether this finding is due to an increase in *Treponema* or a decrease in *Corynebacterium*, but the ratio of these two organisms is a consistent biomarker of periodontal status.

To validate the robustness of the MIP, we tested the ability of the MIP to classify samples from shallow versus deep periodontal pockets. When using the entire dataset (1832 ASVs), samples were classified with an accuracy of 0.88 ± 0.04. When using just the subset of data used to generate the MIP (164 ASVs, or less than 10% of all ASVs), samples were classified with an accuracy of 0.83 ± 0.04% (Fig. 3C). To determine whether the MIP had significantly different accuracy compared to the whole dataset, stratified k-fold cross-validation with a 50:50 train and test set split was repeated 10-fold with random shuffling. On each split, a Random Forests classifier was trained and tested using the whole table versus the table filtered only for *Treponema* and *Corynebacterium* taxa, and the difference in the model performance was tested for significance on the contingency table between classifiers using a McNemar’s test^23^ (Table S3). Additionally, each fold classifier was evaluated by the ROC AUC between classifiers (Fig. 3C). In both evaluations, only a one-fold split showed a significant difference by the classifier, and the mean ROC AUC differed by only 5%. This demonstrates that the classification of the disease state is almost as accurate for *Treponema* and *Corynebacterium* measurements alone as all taxa.

Other microbial indicators of periodontitis have been proposed based on the analysis of chronic periodontitis^24,25^. We assessed these alternative microbial ratios from Chen et al.^24^, (*Treponema denticola, Mogibacterium timidum, Fretibacterium spp*., and *Tannerella forsythia* vs. *Actinomyces naeslundii* and *Streptococcus sanguini*s; representing 158 vs 238 ASVs, respectively), and Meuric et al.^25^, (*Eubacterium, Campylobacter, Treponema*, and *Tannerella* vs. *Veillonella, Neisseria, Rothia, Corynebacterium*, and *Actinomyces*; representing 27 vs. 9 ASVs, respectively) in this dataset. Both of these microbial ratios significantly discriminated healthy from diseased subgingival plaque samples. Random Forests classification with McNemar significance testing as described above showed no significant improvement of these alternative taxa ratios for discriminating between healthy versus diseased samples over the more focused *Treponema*:*Corynebacterium* MIP. We focused on the *Treponema*:*Corynebacterium* MIP as a proxy for early microbial dysbiosis to determine its association with physiological parameters of early periodontitis and cardiovascular disease.

Interestingly, even in subgingival plaque samples from shallow sites (*n* = 779), the MIP was significantly associated with the percent of sites bleeding on probing across the whole mouth (Pearson correlation = 0.243, *p*-value = 8.06e^−12^) (Fig. 3D). This indicates that even before clinically meaningful pocketing develops; there are microbial changes in the subgingival plaque related to gingival inflammation.

### Evaluating the MIP in saliva

**Fig. 4:**
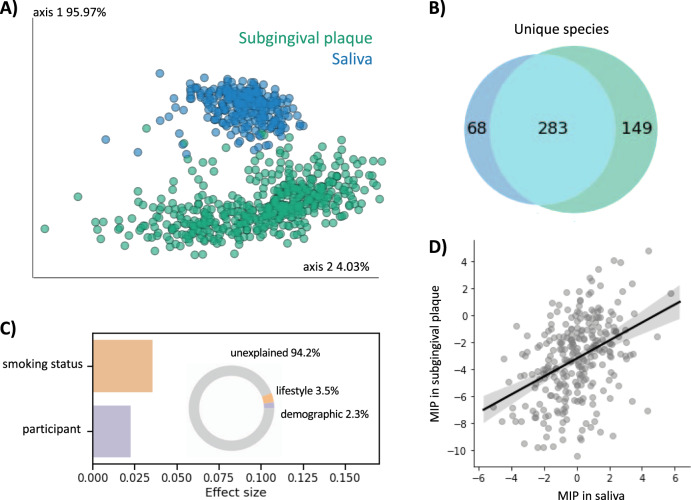
Plaque and saliva are compositionally distinct but have correlated MIP. **A** Beta-diversity analysis with RPCA shows distinct clustering of saliva vs. subgingival plaque samples (PERMANOVA < 0.001). **B** Venn diagram of 16 S rRNA gene amplicon sequencing data collapsed to the species level shows a majority of microbial species were identified in both saliva and subgingival plaque, and that subgingival plaque was more diverse. **C** Redundancy analysis (RDA) estimates the percent microbial diversity explained by each variable. Inset donut chart sums effect sizes by category; unlike subgingival plaque, saliva microbial diversity is driven by lifestyle or demographic variables and is not significantly explained by metabolic or periodontal measures. **D** Microbial indicator of periodontal disease (MIP) was significantly correlated between subgingival plaque and saliva samples, despite having been processed at different institutes with different sequencing parameters and being related to different variables (Pearson *R* = 0.387, *p*-value = 3.97E-11).

Collection of subgingival plaque is not trivial and requires clinically trained professionals. Saliva requires less time and participant burden to collect and can be done remotely. We performed 16 S rRNA gene amplicon sequencing on a subset of saliva samples (*n* = 282) collected in parallel to the subgingival plaque samples from the same individuals. Because the saliva and subgingival plaque samples were processed with different sequencing strategies at different institutions, we first assigned taxonomy to the Human Oral Microbiome Database (HOMD), a well-curated database of full-length 16 S rRNA gene amplicon sequences found in the human oral cavity^26^. We collapsed the subgingival plaque and saliva datasets to the species level and merged the tables together. Beta-diversity analysis of the merged table revealed that, as suspected from previous research, saliva and subgingival plaque had compositionally distinct microbial communities (Fig. 4A). The majority of microbial taxa in the merged table were found in both subgingival plaque and saliva, although each niche also contained distinct microbiota, with subgingival plaque being more diverse than saliva (Fig. 4B).

Effect size analysis using RDA of just the saliva table revealed different factors that drive microbial diversity in saliva compared to subgingival plaque (Fig. 4C). Eight factors were included in the RDA, including two demographic factors (participant and age), three metabolic factors (average systolic blood pressure, BMI, and prediabetes status), two periodontal factors (average whole-mouth attachment loss and average whole-mouth periodontal pocket depth), and one lifestyle factor (tobacco-smoking status). Overall, the percent explained was much lower in saliva compared to subgingival plaque (5.8% versus 22.3%, respectively). The only significant factors in the saliva RDA were smoking status and participant (Fig. 4C). Despite clear differences in microbial community composition between saliva and subgingival plaque, the MIP was significantly correlated (Pearson *R* = 0.387, *p*-value = 3.97E^−11^) (Fig. 4D).

### MIP and periodontal measures

We assessed the correlation of the MIP with various whole-mouth periodontal measures. Subgingival plaque MIP was positively, significantly correlated with the percent of sites bleeding on probing, mean full-mouth pocket depth, and mean full-mouth attachment loss (Table 1). Subgingival plaque MIP was also very highly correlated with Faith’s phylogenetic diversity, a measurement of alpha diversity that accounts for phylogenetic relatedness^27^. All of these correlations held true when looking at only samples from healthy sites, again suggesting that microbial community shifts precede detectable disease (Table S4).

**Table 1:** Microbial indicator of periodontal disease (MIP) in subgingival plaque (*n* = 787) and multiple measures of periodontal disease.

	*N*	Mean MIP parameter estimate	Standard error	*p*-value
Attachment Loss	671	0.032	0.0095	**0.001**
Pocket Depth	671	0.035	0.0051	**<0.0001**
%BOP	670	0.014	0.0025	**<0.0001**
Faith_pd	678	0.65	0.031	**<0.0001**

Mean subgingival plaque was calculated if participant supplied both a diseased and healthy site. All models control for age, sex, race, BMI and smoking status. Attachment Loss average attachment loss. Pocket Depth average periodontal pocket depth, % BOP percent of sites bleeding on probing, Faith_pd Faith’s phylogenetic diversity. Samples lacking even a single read count aligning to either the genus *Treponema* or *Corynebacterium* were dropped (since the logarithm of zero is undefined). Bolded values represent statistically significant Pearson correlations for linear trend (*p* < 0.05).

Saliva MIP was significantly correlated with average full-mouth pocket depth, but not percent of sites bleeding on probing or attachment loss (Table 2). This held true when looking only at participants with moderate or severe periodontitis but not healthy participants, suggesting that microbial changes in the subgingival pocket precede microbial changes in saliva (Table S5). Saliva MIP was also strongly correlated with Faith’s phylogenetic diversity in all patients.

**Table 2:** Microbial indicator of periodontal disease (MIP) in saliva (*n* = 282) and multiple measures of periodontal disease.

	*N*	Mean MIP parameter estimate	Standard error	*p*-value
Attachment Loss	217	0.011	0.0292	0.71
Pocket Depth	217	0.053	0.0155	**0.001**
%BOP	217	0.010	0.0056	**0.07**
Faith_pd	208	0.80	0.097	**<0.0001**

All models control for age, sex, race, BMI, and smoking status. Attachment Loss average attachment loss. Pocket Depth average periodontal pocket depth, %BOP percent of sites bleeding on probing, Faith_pd Faith’s phylogenetic diversity. Samples lacking even a single read count aligning to either the genus *Treponema* or *Corynebacterium* were dropped (since the logarithm of zero is undefined). Bolded values represent statistically significant Pearson correlations for linear trend (*p* < 0.05).

### MIP and metabolic metrics

The overarching goal of the ORIGINS project is to identify associations between oral microbes, oral health, and cardiometabolic health. To this end, we evaluated the correlation of the MIP with various cardiometabolic health metrics. Subgingival plaque MIP was positively, significantly correlated with average systolic and diastolic blood pressure, fasting insulin levels and HOMA-IR, after adjusting for age, sex, race, BMI, and smoking status (Table 3). Together these results suggest that the microbial changes underlying periodontal health are also associated with cardiometabolic health.

**Table 3:** Microbial indicator of periodontal disease (MIP) in subgingival plaque (*n* = 787) is correlated with markers of cardiometabolic health.

	*N*	Mean MIP parameter estimate	Standard error	*p*-value
Meansbp	678	0.41	0.159	**0.01**
Meandbp	678	0.36	0.127	**0.01**
Glucosecrc	676	0.070	0.1122	0.53
Insulin	667	0.14	0.065	**0.03**
HOMA-IR	676	0.0004	0.00016	**0.02**
HbA1c	678	0.003	0.0065	0.60

All models control for age, sex, race, BMI, and smoking status. Mean subgingival plaque was calculated if the participant supplied both a diseased and healthy site. Meansbp mean systolic blood pressure, meandbp mean diastolic blood pressure, glucosecrc fasting glucose, fasting insulin, HOMA-IR Homeostatic Model Assessment for Insulin Resistance measurement, HbA1C hemoglobin A1c. Bolded values represent statistically significant Pearson correlations for linear trend (*p* < 0.05).

Saliva MIP was not significantly correlated with cardiometabolic metrics after adjusting for age, sex, race, BMI, and smoking status (Table 4).

**Table 4:** Microbial indicator of periodontal disease (MIP) in saliva (*n* = 282) is not correlated with markers of cardiometabolic health.

	*N*	Mean MIP parameter estimate	Standard error	*p*-value
Meansbp	217	0.13	0.492	0.80
Meandbp	217	0.18	0.374	0.64
Glucosecrc	215	−0.16	0.317	0.62
Insulin	217	0.007	0.1826	0.97
HOMA-IR	215	−0.00007	0.00045	0.88
HbA1c	217	−0.009	0.0262	0.73

All models control for age, sex, race, BMI, and smoking status. Meansbp mean systolic blood pressure, meandbp mean diastolic blood pressure, glucosecrc fasting glucose, fasting insulin, HOMA-IR Homeostatic Model Assessment for Insulin Resistance measurement, HbA1C hemoglobin A1c. Bolded values represent statistically significant Pearson correlations for linear trend (*p* < 0.05).

### Quartiles of MIP and metabolic metrics and periodontal measures

We evaluated the correlation of quartiles of subgingival plaque MIP with various periodontal measures and cardiometabolic health metrics (Table 5). As quartiles of subgingival plaque MIP increase, mean full-mouth attachment loss, mean full-mouth pocket depth, percent of sites bleeding on probing, and Faith’s phylogenetic diversity all increased significantly even after adjusting for age, sex, race, BMI, and smoking status. Similarly, average systolic and diastolic blood pressure, fasting insulin levels, and HOMA-IR are positively, significantly correlated with quartiles of MIP. In addition, we evaluated the correlation of quartiles of saliva MIP with various periodontal measures and cardiometabolic health metrics and (Table 6). Quartiles of saliva MIP were only positively, significantly correlated with mean full-mouth pocket depth and Faith’s phylogenetic diversity after adjusting for age, sex, race, BMI, and smoking status.

**Table 5:** Quartiles of Microbial Indicator of Periodontal Disease (MIP) in subgingival plaque and multiple measures of periodontal disease and markers of cardiometabolic health.

	MIP Quartile 1 (*N* = 258)	MIP Quartile 2 (*N* = 259)	MIP Quartile 3 (*N* = 259)	MIP Quartile 4 (*N* = 259)	*p*-value
Attachment Loss	*N* = 257 0.53 ± 0.039	*N* = 255 0.54 ± 0.039	*N* = 256 0.62 ± 0.039	*N* = 257 0.71 ± 0.040	**0.001**
Probing Depth	*N* = 257 1.82 ± 0.021	*N* = 255 1.87 ± 0.021	*N* = 256 1.93 ± 0.021	*N* = 257 2.03 ± 0.021	<**0.0001**
%BOP	*N* = 257 0.27 ± 0.01	*N* = 254 0.29 ± 0.01	*N* = 256 0.34 ± 0.01	*N* = 257 0.35 ± 0.01	<**0.0001**
Faith_pd	*N* = 258 11.2 ± 0.13	*N* = 259 13.0 ± 0.12	*N* = 259 14.8 ± 0.12	*N* = 259 16.1 ± 0.13	<**0.0001**
Meansbp	*N* = 258 116.6 ± 0.66	*N* = 257 116.4 ± 0.66	*N* = 257 117.7 ± 0.66	*N* = 257 118.2 ± 0.66	**0.01**
Meandbp	*N* = 258 71.8 ± 0.53	*N* = 257 72.1 ± 0.52	*N* = 257 73.3 ± 0.53	*N* = 257 73.5 ± 0.53	**0.003**
Glucosecrc	*N* = 257 84.9 ± 0.46	*N* = 259 84.6 ± 0.46	*N* = 258 84.7 ± 0.46	*N* = 258 85.0 ± 0.46	0.28
Insulin	*N* = 258 7.5 ± 0.28	*N* = 259 7.8 ± 0.27	*N* = 259 8.2 ± 0.27	*N* = 259 8.3 ± 0.28	**0.04**
HOMA-IR	*N* = 257 0.016 ± 0.001	*N* = 259 0.016 ± 0.001	*N* = 258 0.017 ± 0.001	*N* = 258 0.018 ± 0.001	**0.02**
HbA1c	*N* = 258 5.28 ± 0.027	*N* = 259 5.27 ± 0.027	*N* = 259 5.26 ± 0.027	*N* = 259 5.28 ± 0.027	0.97

Values represent the mean ± the standard error of the mean. Linear mixed models were used in order to control for within Pearson correlation. All models control for age, sex, race, BMI, and smoking status. Attachment Loss average attachment loss, Pocket Depth average periodontal pocket depth, %BOP percent of sites bleeding on probing; meansbp mean systolic blood pressure, meandbp mean diastolic blood pressure, glucosecrc fasting glucose, fasting insulin, HOMA-IR homeostatic model assessment of insulin resistance, HbA1C hemoglobin A1c, Bolded values represent statistically significant Pearson correlations for linear trends (*p* < 0.05).

**Table 6:** Quartiles of Microbial Indicator of Periodontal Disease (MIP) in saliva and multiple measures of periodontal disease and markers of cardiometabolic health.

	MIP Quartile 1 (*N* = 70)	MIP Quartile 2 (*N* = 74)	MIP Quartile 3 (*N* = 68)	MIP Quartile 4 (*N* = 70)	*p*-value
Attachment Loss	*N* = 52 1.05 ± 0.093	*N* = 54 0.91 ± 0.095	*N* = 50 0.85 ± 0.092	*N* = 53 1.00 ± 0.095	0.6177
Probing Depth	*N* = 52 1.72 ± 0.050	*N* = 54 1.81 ± 0.051	*N* = 50 1.85 ± 0.050	*N* = 53 1.87 ± 0.051	**0.0305**
%BOP	*N* = 52 0.26 ± 0.018	*N* = 54 0.26 ± 0.018	*N* = 50 0.29 ± 0.017	*N* = 53 0.27 ± 0.018	0.3702
Faith_pd	*N* = 51 12.08 ± 0.314	*N* = 54 13.10 ± 0.324	*N* = 50 14.43 ± 0.312	*N* = 53 15.28 ± 0.322	<**0.0001**
Meansbp	*N* = 52 119.2 ± 1.55	*N* = 54 121.7 ± 1.59	*N* = 50 119.2 ± 1.55	*N* = 53 119.2 ± 1.60	0.7583
Meandbp	*N* = 52 74.2 ± 1.18	*N* = 54 76.2 ± 1.21	*N* = 50 74.1 ± 1.18	*N* = 53 74.8 ± 1.22	0.9893
Glucosecrc	*N* = 52 88.9 ± 1.01	*N* = 54 86.0 ± 1.02	*N* = 50 86.8 ± 0.99	*N* = 51 87.3 ± 1.03	0.3732
Insulin	*N* = 52 7.97 ± 0.576	*N* = 54 7.97 ± 0.589	*N* = 50 7.96 ± 0.574	*N* = 53 7.97 ± 0.593	0.9995
HOMA-IR	*N* = 52 0.018 ± 0.0014	*N* = 54 0.016 ± 0.0014	*N* = 50 0.017 ± 0.0014	*N* = 51 0.017 ± 0.0014	0.8211
HbA1c	*N* = 52 5.35 ± 0.084	*N* = 54 5.39 ± 0.086	*N* = 50 5.35 ± 0.083	*N* = 53 5.24 ± 0.086	0.3920

Values represent the mean ± the standard error of the mean. Linear mixed models were used in order to control for within Pearson correlation. All models control for age, sex, race, BMI, and smoking status. Attachment Loss average attachment loss, Pocket Depth average periodontal pocket depth, %BOP percent of sites bleeding on probing, meansbp mean systolic blood pressure, meandbp mean diastolic blood pressure, glucosecrc fasting glucose, fasting insulin, HOMA-IR homeostatic model assessment of insulin resistance, HbA1C hemoglobin A1c. Bolded values represent statistically significant Pearson correlations for linear trends (*p* < 0.05).

## Discussion

In a cohort of 787 healthy individuals, we were able to identify early microbial markers of periodontal disease. Microbial composition in subgingival plaque was most strongly explained by periodontal metrics such as subgingival pocket depth and percent of sites bleeding on probing. Both RPCA beta-diversity analysis and redundancy analysis (RDA) showed that the microbial composition in periodontal plaque was more similar between different individuals with the same periodontal phenotype (i.e., shallow or deep pockets), compared to plaque from a shallow versus deep periodontal site within the same person. This finding replicates prior publications showing similar patterns. Since previous studies have shown increased microbial burden in subgingival pockets with periodontitis^28^, it is likely that microbial load varied greatly across the samples in this dataset, and therefore, it is crucial to use scale-invariant analyses. This point is underscored by the fact that this result was not observed using metrics that do not account for compositionality (e.g., UniFrac, Bray–Curtis), which can be greatly affected by microbial load^21^.

RDA further revealed that saliva microbial communities were influenced by different factors compared to subgingival plaque. For instance, while tobacco smoking did not have a significant effect size in subgingival plaque microbial composition, it had the biggest effect size in saliva microbial composition. This is in line with previous reports showing that microbial composition in oral washes was affected by smoking status^29^, while subgingival plaque is not greatly affected by smoking status^30^. Together, these results demonstrate that saliva and subgingival plaque microbial communities are driven by different environmental factors.

We used the factor with the highest effect size on microbial diversity in subgingival plaque, whether the sample came from a deep or shallow pocket, to identify a microbial indicator of early periodontal disease. Using reference frames, we calculated the log-ratio of *Treponema*:*Corynebacterium* and found that it significantly differentiated healthy from diseased periodontal pocket sites. This log-ratio was used as a MIP. Previous studies have proposed alternative MIPs^24,25^, which were also significantly different between health and disease in this dataset, but were not statistically more predictive than the *Treponema:Corynebacterium* ratio for discriminating disease type. We must acknowledge that identifying microbial biomarkers in next-generation sequencing datasets always carries the risk for false positives, but in this study, we focused on the *Treponema* and *Corynebacterium* ASVs because the ability of all the species and strains of these genera to significantly discriminate sites of early disease before clinically meaningful deep pockets formed suggests that these phylogenetic branches have been evolutionarily conserved in subgingival plaque biofilm formation.

Subgingival plaque MIP was significantly correlated with poor periodontal health across a wide range of metrics when only looking at healthy plaque samples, suggesting that microbial communities change before the disease is clinically detectable. Red complex organisms canonically associated with periodontitis in the literature were also positively correlated with periodontal disease status, but they were not as widely prevalent across samples which complicates scale-invariant analyses. This is likely due to the fact that periodontitis prevalence was low and the extent and severity of disease was relatively mild and in the early stages compared to many other studies of periodontitis.

Remarkably, phylogenetically informed alpha diversity was also strongly correlated with MIP in both saliva and subgingival plaque across all periodontal status categories. Both *Treponema* and *Corynebacterium* species have been identified as microbial scaffolds in plaque biofilms. In the context of healthy periodontal plaque, reproducible biofilms with a specific taxonomic organization, referred to as ‘hedgehog’ biofilms, are widely prevalent^31^. In the context of severe periodontitis, *Treponema* taxa have been found in the deepest sections of the periodontal pocket, as they are especially sensitive to oxygen compared to *Corynebacterium*, and form close associations with diverse rod-like bacteria^32–34^. In light of our finding that the ratio of *Treponema* to *Corynebacterium* increases in periodontal disease, this suggests that the biofilm structure shifts from being scaffolded primarily by *Corynebacterium* to *Treponema*, where *Treponema* biofilms are more phylogenetically diverse than *Corynebacterium* biofilms.

One outstanding question that emerges from this analysis is whether microbial dysbiosis is the cause or symptom of periodontitis; does the subgingival pocket first get deeper, providing a microbial niche which *Treponema* species are particularly adept at colonizing? Or does *Treponema* colonization and protease release cause subgingival pocket deepening? The data here are suggestive that *Treponema* first colonizes the periodontal pocket and drives disease, because we see the MIP ratio correlating with pocket depth even before the site would be labeled as diseased (i.e., <3 mm depth). Further, Treponema can move through viscous environments, including between and through tissue^35^. However, further longitudinal studies are required to validate this hypothesis.

Despite the fact that saliva has a compositionally distinct microbiome compared to subgingival plaque, is driven by different metadata variables, and was sequenced independently with different parameters, we found that the MIP was significantly correlated between plaque and saliva. Saliva MIP was also correlated with poor periodontal health, although only in participants with moderate to severe periodontitis. Previous work has found that saliva flow shapes microbial organization in plaque^36^, providing a potential explanation for the correlation in microbial dysbiosis in these distinct microbial communities. One limitation of this analysis was that only a subset of samples had paired saliva sequencing, and increasing the number of saliva samples analyzed could further clarify the potential for saliva microbial composition as a readout for periodontal status, as has been previously suggested by smaller-scale studies^37–39^.

Periodontal disease and cardiometabolic health have been found to co-associate across diverse populations^40,41^. This association is potentially bidirectional. On the one hand, poor cardiovascular health has been suggested to increase the risk of periodontitis^42^, where factors like dysglycemia, receptor for advanced glycation end-product activity and immunological response could contribute to disease. On the other hand, microbial dysbiosis common in periodontitis has been suggested as a risk factor for cardiometabolic disease, since it can evoke persistent inflammation^41,43^. To further explore these associations, we evaluated the correlation of our MIP with various cardiometabolic measurements. We show that specific taxa associated with periodontal disease (the MIP) in subgingival plaque are also significantly correlated with blood pressure, fasting insulin, and HOMA-IR. There was a correlation between subgingival plaque MIP and cardiovascular health markers even in participants with no periodontitis. This suggests that the early origins of these diseases are intricately linked. One potential mechanism could be changed in the enterosalivary nitrate metabolism pathway, which affects systemically available nitric oxide and directly influences cardiometabolic outcomes^44^. For example, *Treponema* colonization and subsequently increased alpha diversity could be leading to depletion of oral nitrate reducers and nitrite depletion. However, any potential cause-and-effect relationship remains to be determined.

Through this large-scale analysis of wave 2 of the ORIGINS cohort^15^, we identified a simple microbial signature based on two common oral taxa associated with periodontal status that also correlates with biomarkers of cardiometabolic disease. Importantly, these microbial community composition transitions appear to occur early in disease before severe periodontitis is evident or clinical cardiovascular disease develops. The results from this analysis suggest that these microbial changes occur first in plaque, and as disease progresses can be identified in saliva. Future longitudinal sampling will allow for more definitive determinations of how the *Treponema:Corynebacterium* ratio is potentially involved in the pathogenesis of periodontal and cardiometabolic diseases.

## Methods

### Sample collection

ORIGINS is an occupation-based cohort study among members of the Service Employees International Union 1199 designed to investigate the relationship between oral microbial community composition and glucose metabolism. Periodontal examination, subgingival plaque, and saliva collection were performed as previously described^15^. In summary, 1188 subgingival plaque samples (4 samples from 297 participants) were collected from the most posterior tooth per quadrant (excluding third molars) via sterile curettes after removal of the supragingival plaque. Unstimulated saliva was collected from each participant in parallel.

### Ethical approval

The Institutional Review Board at Columbia University and the University of Minnesota approved the study protocol. All participants provided written informed consent.

### DNA extraction and 16 S rRNA gene sequencing

DNA was extracted from subgingival plaque and saliva samples at The Forsyth Institute. 16 S rRNA gene amplicon sequencing was performed on subgingival plaque samples by The Forsyth Institute using primers targeting variable regions 3 and 4; Forward- CCTACGGGAGGCAGCAG (341 f) and Reverse- GGACTACHVGGGTWTCTAAT (806r). Sequencing was performed on a MiSeq using a Paired End 250 cycle kit.

16 S rRNA gene amplicon sequencing libraries on DNA extracted from saliva was performed at UC San Diego using the Earth Microbiome Project protocol^45,46^ with primers targeting the v4 region; Forward- GTGYCAGCMGCCGCGGTAA (515 f) and Reverse- GGACTACNVGGGTWTCTAAT (806r). Sequencing was performed on a MiSeq using a Paired End 150 cycle kit.

### Sequence analysis

Raw reads were analyzed with QIIME2. Demultiplexed sequences were quality-filtered with default parameters in qiime quality-filter *q*-score; namely, reads were trimmed after the first appearance of three basecalls with a PHRED score of four or less, and the entire read was removed if the read was truncated to <75% of the input sequence. Quality filtered forward-read sequences were denoised using Deblur^47^ with the default parameters. Samples with less than 1000 quality-filtered reads were removed from downstream analysis. In order to remove reads aligned to chloroplast or mitochondrial genes, sequences were aligned using a classifier pretrained on the GreenGenes v13_8 database with 99% sequence homology using sklearn^48^. Sequences aligned to mitochondria or chloroplast were removed using filter-table --p-exclude (0.005% of the entire dataset). A phylogenetic tree was created using fragment insertion via SEPP^49^. Taxonomy was assigned using sklearn^48^ against the HOMD database version 15.1^26^. All features not present in at least 1% of samples were excluded from downstream analysis.

The final quality-filtered subgingival plaque table contained 43,709,128 reads (97.6% of the raw dataset) across 1107 samples (99.2% of total collected samples) with a total of 1832 amplicon sequence variants (ASV) (26.9% of all identified ASVs), of which 99.5% were assigned to at least the phylum level. The final quality-filtered saliva table contained 4,892,251 (99.6% of the raw dataset) reads across 282 samples (100% of sequenced samples) with a total of 859 ASVs (73.0% of all identified ASVs), of which 99.5% were assigned to at least the phylum level.

### Differential abundance testing

To determine which taxa are associated with which phenotypes in our dataset, we used the concept of Reference Frames^21^. This tool accounts for the compositional nature of next-generation sequencing experiments^14^. In brief, comparing relative abundances among sample groups can be misleading when the total microbial load is unknown, as is the case in this dataset. To avoid these pitfalls, we used the machine learning tool Songbird (https://github.com/biocore/songbird) to perform multinomial regression and then ranked each ASV by its coefficient in the regression model to determine each taxon’s relative differential across a given phenotype. Periodontal pocket depth was used as the formula in the model. The number of random test samples held back for validation in the model was 111 (10%). We used a batch size of ten with 500 epochs (number of passes through the entire dataset to train the model), a learning rate of 0.001 and a differential prior of 10. The resulting ranks (differentials.qza) were visualized with Qurro^22^ and allowed us to prioritize which taxa were most associated with a given phenotype.

To identify taxa associated with shallow versus deep periodontal pockets, we browsed the highest and lowest-ranked microbes in this category using Qurro. ASVs assigned to the genus *Corynebacterium* were mostly associated with shallow pockets, whereas ASVs assigned to the genus *Treponema* were mostly associated with deep pockets. To generate a microbial indicator of periodontitis (MIP), we used *Corynebacterium* as a ‘reference frame’ and calculated the log ratio of all *Treponema* counts to all *Corynebacterium* counts.

### Classification

A Random Forests (Breiman 2001) (RF) model was trained to predict disease status based on shallow (pocket depth <3 mm) versus deep (pocket depth > 4 mm) periodontal pockets. The RF model was trained using a Stratified K-Folds cross-validation (CV) with 10-Fold CV splits. On each CV split, an RF model with 500 estimators was trained, and RF probability predictions were compared to the test set using the Receiver Operating Characteristic (ROC). The mean and standard deviation from the mean were calculated for the Area Under the Curve (AUC) across the 10-fold CV. This classification was performed on the whole ASV level data table and compared to the table filtered for only members of *Treponema* and *Corynebacterium* concatenated with the log-ratio of *Treponema* to *Corynebacterium*. All classification was performed through Scikit-learn (v. 0.22.2)^48^. To determine statistical significance, stratified k-fold cross-validation with a 50:50 train and test set split was repeated 10-fold with random shuffling. On each split, a Random Forests classifier was trained and tested using the whole table, versus the table filtered only for *Treponema* and *Corynebacterium* taxa, and the difference in the model performance was tested for significance on the contingency table between classifiers using a McNemar’s test^23^.

### Reporting Summary

Further information on research design is available in the Nature Research Reporting Summary linked to this article.

## Supplementary information

Reporting Summary Checklist

Table S1

Table S2

Table S3

table S4

Table S5

## Data Availability

All sequencing data and sample metadata are available through Qiita^50^ under study ID 11808 for saliva data and study ID 14375 for subgingival plaque samples. Raw sequence data is also available through EBI accession PRJEB50306 for saliva samples and accession PRJEB50261 for subgingival plaque samples.

## References

[1] Xu X (2015). Oral cavity contains distinct niches with dynamic microbial communities. Environ. Microbiol..

[2] Abiko Y, Sato T, Mayanagi G, Takahashi N (2010). Profiling of subgingival plaque biofilm microflora from periodontally healthy subjects and from subjects with periodontitis using quantitative real-time PCR. J. Periodontal Res..

[3] Heller D, Silva-Boghossian CM, do Souto RM, Colombo APV (2012). Subgingival microbial profiles of generalized aggressive and chronic periodontal diseases. Arch. Oral. Biol..

[4] Kageyama S (2017). Relative abundance of total subgingival plaque-specific bacteria in salivary microbiota reflects the overall periodontal condition in patients with periodontitis. PloS One.

[5] Socransky SS, Haffajee AD, Cugini MA, Smith C, Kent RL (1998). Microbial complexes in subgingival plaque. J. Clin. Periodontol..

[6] Nazir MA (2017). Prevalence of periodontal disease, its association with systemic diseases and prevention. Int J. Health Sci..

[7] Meyer MS, Joshipura K, Giovannucci E, Michaud DS (2008). A review of the relationship between tooth loss, periodontal disease, and cancer. Cancer Causes Control.

[8] Könönen E, Gursoy M, Gursoy UK (2019). Periodontitis: a multifaceted disease of tooth-supporting tissues. J. Clin. Med..

[9] Preshaw PM (2012). Periodontitis and diabetes: a two-way relationship. Diabetologia.

[10] Kebschull M, Demmer RT, Papapanou PN (2010). “Gum bug, leave my heart alone!”—epidemiologic and mechanistic evidence linking periodontal infections and atherosclerosis. J. Dent. Res.

[11] Lalla E, Papapanou PN (2011). Diabetes mellitus and periodontitis: a tale of two common interrelated diseases. Nat. Rev. Endocrinol..

[12] Lockhart PB (2012). Periodontal disease and atherosclerotic vascular disease: does the evidence support an independent association?: a scientific statement from the American Heart Association. Circulation.

[13] Emerging Risk Factors Collaboration. Diabetes mellitus, fasting blood glucose concentration, and risk of vascular disease: a collaborative meta-analysis of 102 prospective studies. *Lancet Lond. Engl.* **375**, 2215–2222 (2010). 10.1016/S0140-6736(10)60484-9 PMC2904878 20609967

[14] Gloor GB, Macklaim JM, Pawlowsky-Glahn V, Egozcue JJ (2017). Microbiome datasets are compositional: and this is not optional. Front. Microbiol..

[15] Demmer RT (2015). Periodontal bacteria and prediabetes prevalence in ORIGINS: the oral infections, glucose intolerance, and insulin resistance study. J. Dent. Res.

[16] Martino C. et al. A novel sparse compositional technique reveals microbial perturbations. *mSystems*10.1128/mSystems.00016-19 (2019). 10.1128/mSystems.00016-19 PMC6372836 30801021

[17] Lozupone C, Knight R (2005). UniFrac: a new phylogenetic method for comparing microbial communities. Appl Environ. Microbiol..

[18] Chen C (2018). Oral microbiota of periodontal health and disease and their changes after nonsurgical periodontal therapy. ISME J..

[19] Shi M (2018). The subgingival microbiome of periodontal pockets with different probing depths in chronic and aggressive periodontitis: a pilot study. Front Cell Infect. Microbiol..

[20] Falony G (2016). Population-level analysis of gut microbiome variation. Science.

[21] Morton JT (2019). Establishing microbial composition measurement standards with reference frames. Nat. Commun..

[22] Fedarko MW (2020). Visualizing’omic feature rankings and log-ratios using Qurro. NAR Genom. Bioinform..

[23] McNemar Q (1947). Note on the sampling error of the difference between correlated proportions or percentages. Psychometrika.

[24] Chen T, Marsh PD, Al-Hebshi NN (2021). SMDI: an index for measuring subgingival microbial dysbiosis. J. Dent. Res.

[25] Meuric V (2017). Signature of microbial dysbiosis in periodontitis. Appl. Environ. Microbiol..

[26] Escapa IF (2018). New insights into human nostril microbiome from the expanded human oral microbiome database (eHOMD): a resource for the microbiome of the human aerodigestive tract. mSystems.

[27] Faith DP, Baker AM (2006). Phylogenetic diversity (PD) and biodiversity conservation: some bioinformatics challenges. Evol. Bioinform..

[28] Nastych O (2020). Comparison of bacterial load parameters in subgingival plaque during peri-implantitis and periodontitis using the RT-PCR method. Acta Stomatol Croat..

[29] Wu J (2016). Cigarette smoking and the oral microbiome in a large study of American adults. ISME J..

[30] Lanza E (2016). Complementary clinical effects of red complex bacteria on generalized periodontitis in a caucasian population. Oral. Dis..

[31] Mark Welch JL, Rossetti BJ, Rieken CW, Dewhirst FE, Borisy GG (2016). Biogeography of a human oral microbiome at the micron scale. Proc. Natl Acad. Sci. USA.

[32] Wecke J (2000). A novel technique for monitoring the development of bacterial biofilms in human periodontal pockets. FEMS Microbiol Lett..

[33] Chan ECS, Vries J, de, Harvey RF, Tam Y-C (2011). Use of fluorescence microscopy for monitoring periodontal disease state. Can. J. Microbiol.

[34] Armitage GC, Dickinson WR, Jenderseck RS, Levine SM, Chambers DW (1982). Relationship between the percentage of subgingival spirochetes and the severity of periodontal disease. J. Periodontol..

[35] Radolf JD (2016). Treponema pallidum, the syphilis spirochete: making a living as a stealth pathogen. Nat. Rev. Microbiol.

[36] Proctor DM (2018). A spatial gradient of bacterial diversity in the human oral cavity shaped by salivary flow. Nat. Commun..

[37] Sun X (2020). Alteration of salivary microbiome in periodontitis with or without type-2 diabetes mellitus and metformin treatment. Sci. Rep..

[38] Sabharwal A (2019). The salivary microbiome of diabetic and non-diabetic adults with periodontal disease. J. Periodontol..

[39] Lin M (2020). Saliva microbiome changes in patients with periodontitis with and without chronic obstructive pulmonary disease. Front Cell Infect. Microbiol..

[40] Zhao M-J (2019). Periodontal disease is associated with increased risk of hypertension: a cross-sectional study. Front. Physiol..

[41] Muñoz Aguilera E (2020). Periodontitis is associated with hypertension: a systematic review and meta-analysis. Cardiovasc. Res..

[42] Koo HS, Hong SM (2018). Prevalence and risk factors for periodontitis among patients with metabolic syndrome. Metab. Syndr. Relat. Disord..

[43] Czesnikiewicz-Guzik M (2019). Causal association between periodontitis and hypertension: evidence from Mendelian randomization and a randomized controlled trial of non-surgical periodontal therapy. Eur. Heart J..

[44] Goh CE (2019). Association between nitrate-reducing oral bacteria and cardiometabolic outcomes: results from ORIGINS. J. Am. Heart Assoc..

[45] Marotz C (2017). DNA extraction for streamlined metagenomics of diverse environmental samples. BioTechniques.

[46] Thompson LR (2017). A communal catalogue reveals Earth’s multiscale microbial diversity. Nature.

[47] Amir A (2017). Deblur rapidly resolves single-nucleotide community sequence patterns. mSystems.

[48] Pedregosa F. et al. Scikit-learn: Machine learning in Python. *arXiv*10.48550/arXiv.1201.0490 (2018).

[49] Janssen S (2018). Phylogenetic placement of exact amplicon sequences improves associations with clinical information. mSystems.

[50] Gonzalez A (2018). Qiita: rapid, web-enabled microbiome meta-analysis. Nat. Methods.

