# Supplementary material for: Early microbial markers of periodontal and cardiometabolic diseases in ORIGINS
Source: NPJ Biofilms Microbiomes. 2022 Apr 20;8:30. doi: 10.1038/s41522-022-00289-w (PMC9021254; doi:10.1038/s41522-022-00289-w)
Supplement: Supplementary file 4 — Table S3 [file 41522_2022_289_MOESM4_ESM.docx]

**Supplementary table 3**. McNemar test statistics comparing ROC curves from Figure 3C.

| N-fold | test-statistic | P-value |
| --- | --- | --- |
| 0 | 1 | 0.375 |
| 1 | 3 | 0.508 |
| 2 | 2 | 1.000 |
| 3 | 3 | 0.344 |
| 4 | 2 | 0.109 |
| 5 | 1 | 0.219 |
| 6 | 2 | 0.688 |
| 7 | 4 | 0.754 |
| 8 | 2 | 0.022 |
| 9 | 4 | 1.000 |
