# Supplementary material for: Early microbial markers of periodontal and cardiometabolic diseases in ORIGINS
Source: NPJ Biofilms Microbiomes. 2022 Apr 20;8:30. doi: 10.1038/s41522-022-00289-w (PMC9021254; doi:10.1038/s41522-022-00289-w)
Supplement: Supplementary file 5 — table S4 [file 41522_2022_289_MOESM5_ESM.docx]

**Supplementary table 4**. Microbial Indicator of Periodontal Disease (MIP) correlation with markers of cardiometabolic health in subgingival plaque subset by disease status (H = no periodontitis, D = mild/severe periodontitis, according to the CDC/AAP definition). Mean subgingival plaque was calculated if participant supplied both a diseased and healthy site (n=787). All models control for age, sex, race, BMI and smoking status. Meansbp = mean systolic blood pressure; meandbp = mean diastolic blood pressure; glucosecrc = fasting glucose; hsinsulin = fasting insulin; HbA1C = hemoglobin A1c. Attachment Loss = average attachment loss. Pocket Depth = average periodontal pocket depth; %BOP = percent of sites bleeding on probing; Faith_pd = Faith’s phylogenetic diversity Bolded values represent statistically significant Pearson correlations for linear trends (p<0.05).

|  | N | Mean MIP Estimate | Standard Error | p-value |
| --- | --- | --- | --- | --- |
| Meansbp | 678 | 0.41 | 0.159 | **0.0103** |
| Meansbp H | 619 | 0.30 | 0.129 | **<.0001** |
| Meansbp D | 334 | 0.25 | 0.227 | 0.2786 |
| Meandbp | 678 | 0.36 | 0.127 | **0.0053** |
| Meandbp H | 619 | 0.21 | 0.108 | 0.0547 |
| Meandbp D | 334 | 0.33 | 0.174 | 0.0556 |
| Glucosecrc | 676 | 0.070 | 0.1122 | 0.5332 |
| Glucosecrc H | 616 | 0.117 | 0.0907 | 0.1966 |
| Glucosecrc D | 334 | -0.008 | 0.1562 | 0.9611 |
| Hsinsulin | 667 | 0.14 | 0.065 | **0.0284** |
| Hsinsulin H | 619 | 0.10 | 0.053 | 0.0703 |
| Hsinsulin D | 334 | 0.07 | 0.096 | 0.4778 |
| HOMA-IR | 676 | 0.0004 | 0.00016 | **0.0219** |
| HOMA-IR H | 616 | 0.0003 | 0.00012 | **0.0213** |
| HOMA-IR D | 334 | 0.0002 | 0.00024 | 0.4563 |
| HbA1c | 678 | 0.003 | 0.0065 | 0.6004 |
| HbA1c H | 619 | 0.010 | 0.0051 | **0.0487** |
| HbA1c D | 334 | -0.016 | 0.0099 | 0.1134 |
| Meanaloss | 671 | 0.032 | 0.0095 | **0.0008** |
| Meanaloss H | 619 | 0.009 | 0.0058 | 0.1133 |
| Meanaloss D | 324 | 0.027 | 0.0158 | 0.0872 |
| Meanpd | 671 | 0.035 | 0.0051 | **<.0001** |
| Meanpd H | 619 | 0.012 | 0.0032 | **0.0002** |
| Meanpd D | 324 | 0.036 | 0.0081 | **<.0001** |
| %BOP | 670 | 0.014 | 0.0025 | **<.0001** |
| %BOP H | 618 | 0.008 | 0.0021 | **0.0002** |
| %BOP D | 324 | 0.014 | 0.0035 | **<.0001** |
| Faith_pd | 678 | 0.65 | 0.031 | **<.0001** |
| Faith_pd H | 619 | 0.51 | 0.027 | **<.0001** |
| Faith_pd D | 334 | 0.74 | 0.041 | **<.0001** |
