# Supplementary material for: Early microbial markers of periodontal and cardiometabolic diseases in ORIGINS
Source: NPJ Biofilms Microbiomes. 2022 Apr 20;8:30. doi: 10.1038/s41522-022-00289-w (PMC9021254; doi:10.1038/s41522-022-00289-w)
Supplement: Supplementary file 6 — Table S5 [file 41522_2022_289_MOESM6_ESM.docx]

**Supplementary table 5**. Microbial Indicator of Periodontal Disease (MIP) correlation with markers of cardiometabolic health in saliva subset by disease status (H = no periodontitis, D = mild/severe periodontitis, according to the CDC/AAP definition). All models control for age, sex, race, BMI and smoking status. Meansbp = mean systolic blood pressure; meandbp = mean diastolic blood pressure; glucosecrc = fasting glucose; hsinsulin = fasting insulin; HbA1C = hemoglobin A1c. Attachment Loss = average attachment loss. Pocket Depth = average periodontal pocket depth; %BOP = percent of sites bleeding on probing; Faith_pd = Faith’s phylogenetic diversity Bolded values represent statistically significant Pearson correlations for linear trends (p<0.05).

|  | **N** | **MIP Estimate** | **Standard Error** | **p-value** |
| --- | --- | --- | --- | --- |
| Meansbp | 217 | 0.13 | 0.492 | 0.7969 |
| Meansbp H | 134 | 0.37 | 0.526 | 0.4784 |
| Meansbp D | 83 | -0.22 | 1.058 | 0.8384 |
| Meandbp | 217 | 0.18 | 0.374 | 0.6380 |
| Meandbp H | 134 | 0.30 | 0.409 | 0.4648 |
| Meandbp D | 83 | 0.05 | 0.802 | 0.9496 |
| Glucosecrc | 215 | -0.16 | 0.317 | 0.6217 |
| Glucosecrc H | 132 | -0.13 | 0.396 | 0.7468 |
| Glucosecrc D | 83 | -0.25 | 0.590 | 0.6678 |
| hsinsulin | 217 | 0.007 | 0.1826 | 0.9685 |
| hsinsulin H | 134 | -0.069 | 0.1960 | 0.7250 |
| hsinsulin D | 83 | -0.004 | 0.3843 | 0.9910 |
| HOMA-IR | 215 | -.00007 | 0.00045 | 0.8809 |
| HOMA-IR H | 132 | -.00027 | 0.00045 | 0.5496 |
| HOMA-IR D | 83 | 0.00013 | 0.00098 | 0.8964 |
| HbA1c | 217 | -0.009 | 0.0262 | 0.7336 |
| HbA1c H | 134 | 0.023 | 0.0312 | 0.4534 |
| HbA1c D | 83 | -0.029 | 0.0508 | 0.5661 |
| Meanaloss | 217 | 0.011 | 0.0292 | 0.7102 |
| Meanaloss H | 134 | -0.019 | 0.0254 | 0.4452 |
| Meanaloss D | 83 | -0.003 | 0.0649 | 0.9582 |
| Meanpd | 217 | 0.053 | 0.0155 | **0.0008** |
| Meanpd H | 134 | 0.002 | 0.0166 | 0.9229 |
| Meanpd D | 83 | 0.105 | 0.0280 | **0.0003** |
| %BOP | 217 | 0.010 | 0.0056 | 0.0681 |
| %BOP H | 134 | 0.002 | 0.0065 | 0.7488 |
| %BOP D | 83 | 0.023 | 0.0112 | **0.0397** |
| Faith_pd | 208 | 0.80 | 0.097 | **<.0001** |
| Faith_pd H | 129 | 0.65 | 0.131 | **<.0001** |
| Faith_pd D | 79 | 1.00 | 0.161 | **<.0001** |
